# EEGIFT: Group Independent Component Analysis for Event-Related EEG Data

**DOI:** 10.1155/2011/129365

**Published:** 2011-06-23

**Authors:** Tom Eichele, Srinivas Rachakonda, Brage Brakedal, Rune Eikeland, Vince D. Calhoun

**Affiliations:** ^1^Department of Biological and Medical Psychology, University of Bergen, Jonas Lies Vei 91, 5011 Bergen, Norway; ^2^Mind Research Network, 1101 Yale Boulevard, N.E, Albuquerque, NM 87131, New Mexico, USA; ^3^Department of Electrical and Computer Engineering, University of New Mexico, Albuquerque, USA

## Abstract

Independent component analysis (ICA) is a powerful method for source separation and has been used for decomposition of EEG, MRI, and concurrent EEG-fMRI data. ICA is not naturally suited to draw group inferences since it is a non-trivial problem to identify and order components across individuals. One solution to this problem is to create aggregate data containing observations from all subjects, estimate a single set of components and then back-reconstruct this in the individual data. Here, we describe such a group-level temporal ICA model for event related EEG. When used for EEG time series analysis, the accuracy of component detection and back-reconstruction with a group model is dependent on the degree of intra- and interindividual time and phase-locking of event related EEG processes. We illustrate this dependency in a group analysis of hybrid data consisting of three simulated event-related sources with varying degrees of latency jitter and variable topographies. Reconstruction accuracy was tested for temporal jitter 1, 2 and 3 times the FWHM of the sources for a number of algorithms. The results indicate that group ICA is adequate for decomposition of single trials with physiological jitter, and reconstructs event related sources with high accuracy.

## 1. Introduction

Event related brain responses to simple cognitive tasks are composed of multiple dynamic, temporally and regionally overlapping, functionally separable sub-processes which add to existing oscillatory background activity [1–5]. In other words, event-related processes are spatially and temporally mixed across the brain, and the scalp EEG samples a volume-conducted, spatially degraded version of the responses, where the potential at any location and latency can be considered a mixture of multiple independent sources that stem from large-scale synchronous field potentials [6, 7]. The exploration of the trial-to-trial variability in these responses provides important clues about the dynamics and adaptability of cognitive processes [2, 6, 8–10] 

One powerful and increasingly popular method that allows for decomposition of EEG data and assessment of single trial variability is blind source separation with independent component analysis (ICA). ICA algorithms solve a two-dimensional linear mixing problem of spatially, and/or temporally independent sources [11, 12]. ICA models spatio-temporal data as a linear combination of maps and timecourses while attempting to maximize the statistical independence between either the maps (spatial ICA, sICA) or the time courses (temporal ICA, tICA). The method has general applicability to Gaussian mixtures, regarding psychophysiological and neuro imaging data it has been successfully used with averaged ERPs [13], single trial EEG [6, 7, 14], structural and functional MRI [15], and recently also in EEG-fMRI integration [16–22]. Tools for data analysis with ICA are implemented for example in the academic freeware toolboxes EEGLAB [23], ICALAB [24] and GIFT [15, 25], both running in Matlab, as well as the stand-alone package FSL-MELODIC [26].

The basic ICA model applies to single subject data, thus one inherent limitation to the use of ICA in typical multi-subject/session EEG studies is that this method is not naturally suited to generalize results from a group of subjects. This is because ICAs from separate runs or participants will generate different sets of components with different order and scaling that need to be matched across datasets to allow group inferences. This is in contradistinction to the straightforward way of making group inferences from, for example, ERP component averages from selected channels and latencies in the general linear model [27, 28]. Therefore, a method combining individual components is desired where group inferences are straightforward. There are two strategies to allow for matching of independent components across individuals: one is to combine individual ICs across subjects with clustering techniques [7, 29–31]. Clustering usually involves selection of suitable algorithms and features of interest, that is, topography, timecourse, spectrum and so forth by which between-subject correspondences of components are identified. This requires additional assumptions about the data and expert user input, and differences in algorithm and feature selection, as well as user bias can then create equivocal results. Alternatively, a more parsimonious approach is to create aggregate data containing observations from all subjects, directly estimate components that are consistently expressed in the population in a single set of ICs and then back-reconstruct estimated components to the individual data. This approach has so far predominantly been used for spatial ICA of fMRI [25, 26, 32]. We have recently adopted a group ICA method for parallel and joint decomposition of concurrent EEG-fMRI recordings [21, 22, 33]. Here, we present a group-level temporal ICA model based on the rationale proposed by Calhoun et al. [25] for single-trial analysis of event related EEG timeseries that we also implement in the toolbox EEGIFT, which is available from the Mind Research Networks' medical image analysis lab webpages at http://icatb.sourceforge.net/gift/eegift_startup.php along with documentation and tutorial datasets. EEGIFT runs in Matlab (The Mathworks, Natick, MA), and employs pre-processed data from EEGLAB [23], a popular free toolbox for EEG processing which can be downloaded from http://sccn.ucsd.edu/eeglab/. 

In order to make the component estimation computationally feasible, we employ a data reduction using principal component analysis (PCA). The number of components estimated from the data can be based on minimum description length [34] principle (MDL) or on other estimates of dimensionality. Due to aggregation and data reduction with PCA preceding component estimation, group ICA of EEG time-domain data is preferentially suited to the detection of components that contribute to event-related potentials. Processes that are not time/phase-locked within and across subjects, such as background rhythms cannot be satisfyingly reconstructed, in these cases the transformation of the data into the frequency domain prior to ICA decomposition is useful [35]. For time domain data it follows that the accuracy of component detection and back-reconstruction with a group model is dependent on the degree of intra- and inter-individual time and phase-locking of event related EEG processes. Similar to findings in early studies of PCA decomposition of ERP averages [36, 37], excess latency jitter results in splitting of a single source into two (or more) independent components representing the source and its approximate time derivative [21]. Here, we illustrate this dependency in a group analysis of 20 hybrid datasets consisting of three simulated event-related sources with varying degrees of latency jitter, mixed with real EEG data from 20 subjects that participated in a passive listening experiment. It is not trivial to recover reliable estimates about ERP latency jitter from real EEG data, we assume from a literature search and unpublished observations in our lab that single trial peak latencies of larger components such as the auditory N1 and P3 vary approximately 20–40 ms^2^, roughly corresponding to the full width at half maximum (FWHM) of these components (for comparison, see, e.g., [8]). We tested the reconstruction accuracy (RA), expressed as the variance of the source accounted for by the reconstructed IC (in terms of *R* ^2^) for latency jitter 1, 2 and 3 times the average FWHM of the sources for the Infomax, ERICA, JADE, fastICA, and SIMBEC algorithms that are implemented among others in GIFT and EEGIFT.

## 2. Method

### 2.1. Group tICA

**Figure 1:**
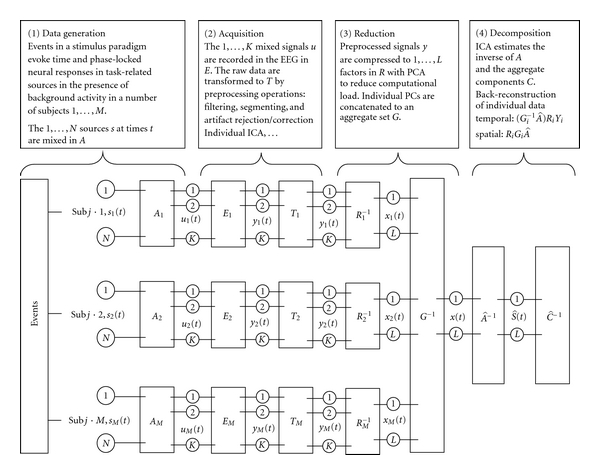
Group ICA. In the group ICA model, we assume that the EEG is a linear mixture of temporally independent sources in each subject *s*(*t*). The linear combination of sources is represented by the unknown mixing matrix *A*, and yields the ideal samples of brain activity u(t), and the signals recorded with the EEG amplifier (*E*). Transformations (*T*) during preprocessing contain filtering, epoching, artefact rejection, individual ICA for additional artefact reduction and so forth, altering the effective temporal sampling and dimensionality of the data *y*(*i*). For each individual separately, the pre-processed single trial data are pre-whitened and reduced to *R* via PCA. Group data is generated by concatenating individual principal components in the aggregate data set *G*. Temporal ICA is performed in this set, estimating aggregate components (*C*). From the aggregate components, the individual data are reconstructed (see text for details).

The group ICA model is divided into the underlying data generation and mixing process, recording, pre-processing, reduction, component estimation and back-reconstruction (schematically illustrated in Figure 1). We assume that the scalp EEG signal is a gaussian mixture containing statistically independent non-gaussian source timeseries *s*(*t*) = [*s* ^1^(*t*),*s* ^2^(*t*),…,*s* ^*N*^(*t*)]^*T*^ indicated by *s*_*i*_(*t*) at time *t* for the *i*th source from *N* sources. The sources have weights that specify the contribution to each timepoint. The weights are multiplied by each source's fixed topography. Secondly, it is assumed that the *N* sources are linearly mixed so that a given time point contains a weighted mixture of the sources. The linear combination of sources is represented by the unknown mixing system *A where As*(*t*) = *u*(*t*) where *u*(*t*) = [*u*_1_(*t*),*u*_2_(*t*),…,*u*_*N*_(*t*)]^*T*^ and represents *N* ideal samples of the signal *u*_*i*_(*t*) at time *t*, for the *i*th source in the brain. The sampling of the electric activity on the scalp results in *y*(*t*) = [*y*_1_(*t*),*y*_2_(*t*),…,*y*_*K*_(*t*)]^*T*^  where the EEG is sampled at *K* timepoints where *t* ∈ {1,2,…, *K*}. A set of possible transformations during pre-processing, such as downsampling and filtering determine the effective sampling such that *y*(*j*) = [*y*_1_(*j*),*y*_2_(*j*),…,*y*_*K*_(*j*)]^*T*^ where the effective temporal sampling is indexed by *j* = 1,2,…, *K*.

### 2.2. Data Reduction

For each individual separately, the pre-processed single trial data *y*(*j*) are pre-whitened and reduced via PCA (Figure 1, *R*_1_^−1^,…, *R*_*M*_^−1^) containing the major proportion of variance in the N uncorrelated timecourses of *x*(*j*) = [*x*_1_(*j*),*x*_2_(*j*),…,*x*_*N*_(*j*)]^*T*^. PCA whitening preconditions the data and simplifies ICA estimation due to the orthogonal projection, reduction of complexity, and de-noising, as well as compressing the data and thus reducing the computational load. Group data is generated by concatenating individual principal components in the aggregate data set *G*. In detail, let *X*_*i*_ = *R*_*i*_^−1^ *Y*_*i*_ be the *L*-*by*-*V* reduced data matrix from subject *i* where *Y*_*i*_ is the *Q*-*by*-*V* data matrix containing preprocessed EEG epochs from all channels, *R*_*i*_^−1^ is the *L*-*by*-*Q* reducing matrix from the principal component decomposition, *V* is the number of timepoints (samples per epoch ∗ trials), *Q* is the number of scalp channels, and *L* is the size of the channel dimension following reduction. The next step is to concatenate the reduced data from all subjects into a matrix and reduce this matrix to *N*, the number of components to be estimated. The *N*-*by*-*V* reduced, concatenated matrix for the *M* subjects is

(1) $$X=G^{-1}\left[\begin{array}{c} R_{1}^{-1}Y_{1}\\ \vdots \\ R_{M}^{-1}Y_{M} \end{array}\right],$$

where *G* ^−1^ is an *N*-*by*-*LM* reducing matrix from a second PCA decomposition and is multiplied on the right by the *LM*-*by*-*V* concatenated data matrix for the *M* subjects.

### 2.3. ICA Estimation

The idea is to find the mixing matrix *A* and compute the *s*_*i*_ sources for the group. After concatenation of individual principal components in the aggregate data set *G*, this matrix is decomposed by *ICA*, estimating the optimal inverse of the mixing matrix $\hat{A}$, and a single set of source timecourses $\left(\hat{S}\right)$. Following *ICA* estimation, we can write $X=\hat{A}\hat{S}$, where $\hat{A}$ is the *N*-*by*-*N* mixing matrix and $\hat{S}$ are the *N*-*by*-*V* component timecourses. Substituting this expression for *X* into (1) and multiplying both sides by *G* results in

(2) $$G\hat{A}\hat{S}=\left[\begin{array}{c} R_{1}^{-1}Y_{1}\\ \vdots \\ R_{M}^{-1}Y_{M} \end{array}\right].$$

### 2.4. Partitioning and Single Subject Reconstruction

Partitioning the matrix *G* by subject provides the following expression

(3) $$\left[\begin{array}{c} G_{1}\\ \vdots \\ G_{M} \end{array}\right]\hat{A}\hat{S}=\left[\begin{array}{c} R_{1}^{-1}Y_{1}\\ \vdots \\ R_{M}^{-1}Y_{M} \end{array}\right].$$

We then write the equation for subject *i* by working only with the elements in partition *i* of the above matrices such that

(4) $$G_{i}\hat{A}{\hat{S}}_{i}=\left[R_{i}^{-1}Y_{i}\right].$$

The matrix ${\hat{S}}_{i}$ in (4) contains the single subject component timecourses for subject *i*, calculated from the following equation

(5) $${\hat{S}}_{i}={\left(G_{i}\hat{A}\right)}^{-1}R_{i}^{-1}Y_{i}.$$

We now multiply both sides of (4) by *R*_*i*_ and write

(6) $$Y_{i}\approx F_{i}G_{i}\hat{A}{\hat{S}}_{i}$$

yielding the ICA decomposition of the data from subject *i* contained in the matrix *Y*_*i*_. The *N*-*by*-*V* matrix ${\hat{S}}_{i}\;$contains the *N* component timecourses, and the *Q*-*by*-*N* matrix $F_{i}G_{i}\hat{A}$ is the single subject mixing matrix, yielding the scalp maps for *N* components.

### 2.5. Generation of Hybrid Data

In this simulation, 20 mutually uncorrelated hybrid EEG datasets were generated containing 63 channels, 256 timepoints and 500 trials. Three event-related sources (S1–S3) with variable topographies across datasets were mixed with real EEG data from 20 participants from an unrelated study. For each single trial, an event related response (ERR) was simulated with two Gaussians (7).

(7) $${\text{ERR}}_{x}=\;a_{1}e^{-{\left(\left(x-b\right)/{3c}_{1}\right)}^{2}}-\frac{2}{3}a_{2}e^{-{\left(\left(x-b\right)/{2c}_{2}\right)}^{2}}.$$

**Figure 2:**
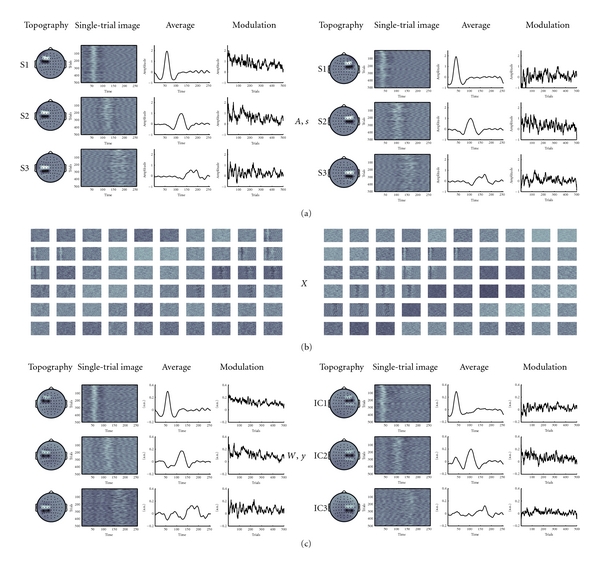
Hybrid Data. The figure shows two hybrid datasets with three source topographies (*A*) and timecourses (*s*) on top, the mixture of the sources with real EEG (*X*) in (*b*), and the reconstruction of the sources after group ICA (*W*, *y*) in (*c*) section.

The amplitudes *a*_1_ and *a*_2_ were varied randomly and independently from 0.5–2.5 and the widths *c*_1_ and *c*_2_ from 0.5–1.5, introducing additional jitter of the ERR amplitude and shape. Latency jitter is a relevant source of variability in single trials, affecting the accuracy of component estimation [21, 36, 37]. The three sources simulated here had non-overlapping peak latencies, and latency variability (within-“subject”) in *b* was in a range of 20 samples in S1, corresponding to the FWHM of the ERR, 40 samples (2 FWHM) in S2, and 60 samples (3 FWHM) in S3. Sine waves with random phase and amplitude modulation were additionally entered as background activity into each source. Across individual datasets the average peak latency of each source dataset was varied by 20 samples (between-“subjects”). For each source, the scalp distributions were generated as dipolar maps covering six channels (of 63), with 50% overlap between S1-S2, and S2-S3, respectively. Across datasets, the location of each source was systematically varied. These sources were normalized to unit variance, and mixed with normalized real EEG data with the same dimensions from 20 participants. The resulting hybrid data are shown in the top half of Figure 2 for two datasets.

### 2.6. Independent Component Analysis

In order to generate a reference value for the performance of group ICA we computed individual ICA solutions in EEGLAB for each of the datasets, employing the Infomax algorithm [38]. For the group ICA, all subjects were analyzed at once, and principal component analysis (PCA) was used for compression to allow the datasets to be processed together. The number of components is estimated by doing singular value decomposition on the data and the resulting eigenvalues are passed to MDL method [34]. Here we selected 20 components as the top 20 components usually explain more than 95% of the variance in the data. In our experience, the exact choice of the number of components does not critically affect the results as long as this number is not much smaller than the true number of sources. In the PCA steps, data from each dataset was reduced over the spatial dimension, that is. from the number of channels to 20 principal components, concatenated across subjects, and again reduced to 20 components. Temporal ICA was then performed using the Infomax algorithm [38] with subsequent back-reconstruction into single subjects.In order to assess reconstruction accuracy of group ICA for different numbers of estimated components, we estimated the solutions for 10, 20, 30, 40 and 50 components using Infomax. For comparison between algorithms, we also estimated solutions using the fastICA [39], JADE [40], SIMBEC [41] and ERICA [42] algorithms. The reconstruction accuracy of group ICA was expressed as the variance of the simulated sources accounted for by the reconstructed ICs averaged across the 20 datasets (*R* ^2^), separately for the entire single trial images, the amplitude modulation across trials around the component peak latency (averaged in a 20 sample window), the component average timeseries, and topographies (Table 1). Results for two hybrid datasets with variable topographic and temporal representations of the three sources are illustrated in Figure 2.

### 2.7. Application to Real Data

In addition to the quantification of the model performance, we illustrate group ICA in the context of typical recording conditions and pre-processing steps that we employ with a decomposition of an auditory oddball dataset. 32 healthy participants took part in the experiment after providing a written statement of informed consent. Participants were sitting in an electro-magnetically shielded and sound-attenuated testing chamber (Rainford EMC Systems, Wigan, UK) and were fitted with 61 Ag/AgCl scalp electrodes mounted in an elastic cap (EasiCap, Falk Minow Services, Breitenbrunn, Germany) and two additional channels monitoring eye movements. All channels were referenced to the nose, and impedances were kept below 10 kΩ. EEGs were recorded continuously at 500 Hz sampling frequency with a band-pass from  .01–250 Hz with BrainAmp DC amplifiers (BrainProducts, Munich, Germany). The experiment consisted of detecting an infrequent target sound within a series of frequent regular sounds and participants were asked to respond as quickly as possible by pressing a button with their right index finger. The standard stimulus was a center-panned 500 Hz tone, the target stimuli were left or right-panned location deviants, respectively. Targets occurred with a probability of 0.1 for each location. Stimulus duration was 75 ms and the inter-stimulus interval was 700 ms, with no immediate repetitions of targets. All stimuli were presented at approximately 65 decibels above threshold. EEGs were down-sampled to 250 Hz, filtered with a zero-phase Butterworth filter from 1–45 Hz, and re-referenced to common average reference. In the example application, the data were segmented from −800 to 1200 ms around target stimuli and thus contain a sequence of standard-target-standard sounds, to dissociate between obligatory stimulus-related and target-related components, respectively. Trials with amplitudes exceeding ±150 *μ*V on any of the channels were excluded from further analysis. Concatenated single sweeps around target onset were subjected to single subject Infomax ICA in EEGLAB [23] running in MATLAB. Components with topographies and timecourses attributeable artifacts were identified and removed from the data [43]. For each dataset, we extract 20 components from the data based on the MDL method. Missing trials were padded with the mean from surrounding trials because there are gradual changes across trials. Single-trials were additionally denoised with a wavelet filter [44]. Hereafter, group ICA was computed, estimating 20 components.

## 3. Results

Reconstruction accuracy (RA) for all analyses is summarized in Table 1. Individual ICA reconstructs the source timecourses with near-perfect accuracy and independent of latency jitter, the topographies are recovered with an accuracy of around 0.9. Group ICA models yield overall lower RA than individual ICA, coming closest to individual ICA in S1, where the entire source timecourse is reconstructed with 0.86 on the average, Infomax with 20 components yields the best performance at 0.88. RA for the amplitude modulation around the peak latency of S1 is 0.9 on the average, for the average timecourses RA is 0.99. These two latter features are typically most relevant for making inferences about electrophysiological data, that is. the overall shape of the waveform and the (single trial) peak amplitudes of components. The topographies of S1 are reconstructed with an accuracy of 0.87. The group ICA result for all features falls with increasing latency jitter regardless of algorithm choice. For the entire timecourse, average RA across algorithms is S1—0.86, S2—0.76, S3—0.50, with the most pronounced drop-off of all features. The peak RA for the peak is S1—0.90, S2—0.87, S3—0.65. RA for the timecourse average is S1—0.95, S2—0.95, S3—0.82, the topographies are reconstructed with S1—0.87, S2—0.80, S3—0.77 overall a less pronounced drop-off. This effect is more pronounced with increasing the number of estimated components as can be seen in the RA of all features of S3. In summary, all three sources were recovered with sufficient reconstruction accuracy of all four features. 

**Figure 3:**
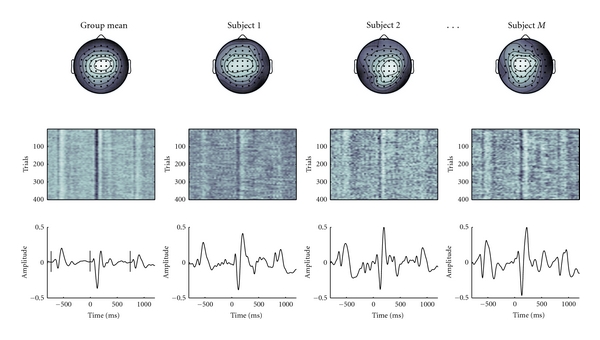
Real Data Decomposition. This figure is an illustration of one component from a group ICA decomposition of an auditory oddball dataset (n = 32 participants), and shows the group mean of one independent component in the leftmost column, and reconstructions for three subject s in the other columns. Note that the group mean component peaks have smaller amplitude due to latency jitter. Top: topography, Middle: single trial image, bottom: component event related average. Vertical lines in the lower left indicate the onset of the preceding standard, target, and succeeding standard, respectively. Topography and latency identify this component as the auditory N1, and typical N1-enhancement is clearly visible in response to targets.

The decomposition of the real data yielded a number of event related components that showed differential responses between standard and target sounds. We related these in terms of the topography and peak latency to the ERP components N1, T-complex, P2, N2 and subcomponents of the P3. In Figure 3, we show the group mean of one independent component that represents the auditory N1, together with the reconstructions for three representative subjects. The group mean of the N1 component is representative for the group level average, and as is expected somewhat smaller in amplitude than compared to a single subject's mean amplitude because of inter-individual latency differences. Parts of this dataset accompany the toolbox as tutorial material and the entire dataset is available upon request.

## 4. Discussion

This work presents an approach to perform a temporal independent component analysis on single-trial time domain EEG data for multiple subjects simultaneously. Our model uses a combination of principal component analysis for data reduction, subsequent independent component analysis on the aggregate data, and back-reconstruction of the aggregate mixing matrix in individual Subjects [20, 25]. This method is implemented in the freeware toolbox EEGIFT that runs in Matlab (R13 and newer) and is downloadable from http://mialab.mrn.org/ or http://icatb.sourceforge.net/. EEGIFT has a graphical user interface (GUI) that allow import of EEG data from multiple participants pre-processed in EEGLAB (http://sccn.ucsd.edu/eeglab/). Another GUI allows the user to specify of analysis details, such as PCA data reduction, model order, choice of ICA algorithm, and the respective parameters. EEGIFT also allow robust estimation with ICASSO [45]. The analysis output is stored after back-reconstruction as individual timecourses and topographies in Matlab format which can be used for specifying between-condition and/or between-groups statistical tests. Individual data, group averages, and population statistics can also be visualized in a GUI as topographies, grand mean timecourses and single trial images. Analyses can also be batch-scripted for convenience. A full documentation of functions in EEGIFT, including a tutorial walkthrough, accompanies the download package (http://icatb.sourceforge.net/gift/eegift_startup.php). User support is provided through the GIFT mailing list (Icatb-discuss). 

In EEGIFT, the back-reconstructed timecourses and topographies are a function primarily of the variability within subjects, as opposed to representing a representation of the average across subjects. A simulation affords to create a situation of a known ground truth with sources and noise parameters, and is useful to illustrate of the concept of the group model. Here, we generated hybrid data with realistic spatial, temporal and amplitude variability. The reconstruction of the solutions with different algorithms and numbers of components demonstrates that this approach offers a straightforward and computationally tractable solution to the problem of multi-subject analysis with ICA. The results from the decomposition of hybrid dataset illustrate that this group model is able to recover timecourses and topographies of event related responses on single trial level with overall sufficiently high accuracy. As laid out in the introduction, the critical determinant for the success of a group model computed for time-domain data is intra- and inter-subject latency jitter. There is considerable difficulty in reliably estimating latency jitter of event related responses in real EEG data; we assume here that physiologic jitter is roughly on the order of the full width at half maximum of sources, in which case the reconstruction accuracy of the group model reaches more than 90% of the accuracy of the individual ICA estimates. Individual ICA with subsequent ordering of components across subject would achieve the highest accuracy with an ideal clustering technique. We did not attempt to directly compare the performance of group-level ICA with that of individual ICA with subsequent clustering, apart from possible computational limitations, the challenge for existing algorithms is to identify and cluster components with poor between-subject correspondence of their topographies and timecourses [7, 29–31]. Group ICA avoids any such problems since the decomposition is computed for all data-sets/subjects simultaneously, estimating a single set of components with the same order across datasets. Consequently, it is straightforward to perform random-effects population tests for the timecourses as well as the topographies. This can also be done in the statistical parametric mapping framework where testing within the Gaussian random field theory with adjustments for multiple comparisons (e.g., [28, 46, 47]). Apart from being used as a primary tool for inference, we believe that there is also a utility for group ICA in the mining stage of prediction-based analysis: For example, in cases where a-priori models of the event related responses can only be poorly specified, for example, where the selection criteria for appropriate electrodes and time-windows from the EEG data are unclear, group ICA results can be used for model specification. 

As noted in Section 1, the limitation of the current method is that responses with poor time/phase-locking are not satisfyingly reconstructed [21, 36, 37]. This is a consequence of the data reduction and aggregation, which inherently limits the visibility of this method to evoked activity, both within and across subjects. Sources that have a loose relation to stimulus/response onset, if extracted and identified, are usually represented in more than one component. This is in clear contradistinction to the superior performance of temporal ICA on concatenated EEG epochs from single subjects, which is insensitive to trial-to-trial phase/latency variability of sources. For detection of poorly time-locked processes in this framework one could consider time-domain data with frequency or time-frequency transformed data [35], or incorporate correction for latency jitter during pre-processing where feasible [8, 44]. We assume from testing the performance of this model extensively in simulated, hybrid, and real data that it is a very useful addition to the available ICA-toolbelt, in that it affords a straightforward possibility to perform group analysis of event related EEG responses. Since the underlying generative model is flexible and modality independent, and the software implementations are highly interrelated, a genuine advantage of this method is that the EEG components can be fused with results from diverse biomedical imaging modalities such as sMRI, fMRI, DTI and VBMas well as genetic information (SNP) within the same conceptual and computational framework [15, 18, 20, 23, 48, 49]. This then affords multimodal inferences which can bear novel insights about brain function.

## Figures and Tables

**Table 1:** Reconstruction Accuracy. The table summarizes the reconstruction accuracy (RA, mean across datasets ± S.E.M.) of different group ICA models. RA stands for proportion of the source variability accounted for by the ICs averaged across the 20 datasets, and was computed separately for the entire single trial images, the amplitude modulation across trials around the component peak latency, the component average timeseries, and topographies.

	Single Trial			Peak Latency			Average			Topography		
Algorithm, Nr IC's	S1	S2	S3	S1	S2	S3	S1	S2	S3	S1	S2	S3
Infomax single, 20	0.98 ± .01	0.97 ± .02	0.98 ± .01	0.99 ± .01	0.99 ± .01	0.99 ± .02	1 ± .00	1 ± .00	1 ± .00	0.89 ± .03	0.89 ± .04	0.90 ± .03
Infomax, 10	0.87 ± .06	0.79 ± .08	0.66 ± .09	0.92 ± .03	0.89 ± .03	0.79 ± .05	0.99 ± .01	0.94 ± .03	0.84 ± .04	0.84 ± .04	0.82 ± .03	0.81 ± .03
Infomax, 20	0.88 ± .04	0.77 ± .07	0.54 ± .08	0.91 ± .03	0.87 ± .03	0.69 ± .06	0.99 ± .01	0.96 ± .02	0.84 ± .05	0.87 ± .04	0.83 ± .05	0.74 ± .05
Infomax, 30	0.87 ± .07	0.76 ± .07	0.50 ± .10	0.91 ± .04	0.86 ± .05	0.65 ± .05	0.99 ± .01	0.95 ± .02	0.86 ± .05	0.87 ± .04	0.81 ± .05	0.83 ± .04
Infomax, 40	0.87 ± .08	0.75 ± .09	0.47 ± .09	0.91 ± .03	0.86 ± .04	0.62 ± .08	0.99 ± .02	0.96 ± .01	0.87 ± .04	0.88 ± .03	0.79 ± .04	0.81 ± .04
Infomax, 50	0.87 ± .05	0.75 ± .06	0.44 ± .11	0.91 ± .05	0.85 ± .04	0.59 ± .07	0.99 ± .01	0.96 ± .01	0.84 ± .05	0.88 ± .04	0.81 ± .05	0.81 ± .04
ERICA, 20	0.88 ± .03	0.73 ± .05	0.46 ± .09	0.92 ± .05	0.85 ± .05	0.62 ± .08	0.99 ± .01	0.89 ± .04	0.77 ± .06	0.86 ± .03	0.76 ± .06	0.71 ± .06
JADE, 20	0.79 ± .06	0.76 ± .07	0.43 ± .10	0.85 ± .03	0.87 ± .05	0.59 ± .09	0.99 ± .02	0.96 ± .02	0.79 ± .06	0.87 ± .05	0.78 ± .08	0.77 ± .07
fastICA, 20	0.86 ± .09	0.77 ± .08	0.53 ± .09	0.90 ± .06	0.87 ± .07	0.68 ± .10	0.99 ± .01	0.96 ± .01	0.82 ± .05	0.87 ± .04	0.84 ± .06	0.74 ± .07
SIMBEC, 20	0.87 ± .08	0.78 ± .08	0.50 ± .09	0.91 ± .06	0.88 ± .06	0.65 ± .07	0.99 ± .01	0.92 ± .03	0.78 ± .06	0.87 ± .03	0.79 ± .04	0.75 ± .05
